# Antioxidant and Immune-Related Implications of Minerals in COVID-19: A Possibility for Disease Prevention and Management

**DOI:** 10.3390/antiox12051104

**Published:** 2023-05-16

**Authors:** Juan M. Toledano, María Puche-Juarez, Jorge Moreno-Fernandez, Julio J. Ochoa, Javier Diaz-Castro

**Affiliations:** 1 Department of Physiology, Faculty of Pharmacy, Campus Universitario de Cartuja, University of Granada, 18071 Granada, Spain; juanmatd97@correo.ugr.es (J.M.T.); jjoh@ugr.es (J.J.O.);; 2 Institute of Nutrition and Food Technology “José Mataix Verdú”, University of Granada, 18071 Granada, Spain; 3 Nutrition and Food Sciences Ph.D. Program, University of Granada, 18071 Granada, Spain; 4 Instituto de Investigación Biosanitaria (IBS), 18016 Granada, Spain

**Keywords:** minerals, COVID-19, SARS-CoV-2, nutrients, immunity, oxidative stress

## Abstract

Since the coronavirus disease 2019 (COVID-19) pandemic appeared, both governments and the scientific community have focused their efforts on the search for prophylactic and therapeutic alternatives in order to reduce its effects. Vaccines against SARS-CoV-2 have been approved and administered, playing a key role in the overcoming of this situation. However, they have not reached the whole world population, and several doses will be needed in the future in order to successfully protect individuals. The disease is still here, so other strategies should be explored with the aim of supporting the immune system before and during the infection. An adequate diet is certainly associated with an optimal inflammatory and oxidative stress status, as poor levels of different nutrients could be related to altered immune responses and, consequently, an augmented susceptibility to infections and severe outcomes derived from them. Minerals exert a wide range of immune-modulatory, anti-inflammatory, antimicrobial, and antioxidant activities, which may be useful for fighting this illness. Although they cannot be considered as a definitive therapeutic solution, the available evidence to date, obtained from studies on similar respiratory diseases, might reflect the rationality of deeper investigations of the use of minerals during this pandemic.

## 1. Introduction

The world is still facing the problems and consequences derived from one of the most relevant public health crises of the last decades. The cluster of pneumonia cases described in China back in 2019 was produced by the severe acute respiratory syndrome coronavirus 2 (SARS-CoV-2). This new virus is the causal agent of the coronavirus disease 2019 (COVID-19), and since its appearance, it has quickly spread to every other country around the world, having been proclaimed by the World Health Organization (WHO) as a global pandemic [1]. This novel pathogen belongs to a type of viruses (β-coronaviruses) frequently found among both birds and mammals. Regarding humans, it is the seventh known coronavirus that can affect them. Despite the variety of previously described coronaviruses, none of them have had such a tremendous impact worldwide as the recently discovered one [2]. The reason for the name of this new virus lays in its genetical similarity to SARS-CoV, responsible for the 2002 outbreak of acute respiratory distress syndrome (ARDS). However, the novelty of SARS-CoV-2 for the immune system and the lack of underlying immunity against this pathogen may be causes of its rapid spread [3].

As with the other coronaviruses, this one is an enveloped single-strained virus, with positive-sense RNA as a genome. It mainly affects the respiratory tract, obtaining access through the droplets produced by an infected subject when they sneeze, cough, or breathe. Although this is the primary route of transmission, the virus can also reach its host through direct contact with a mucus environment, such as nasal fluids or saliva [4]. It accesses cells through the angiotensin-converting enzyme 2 (ACE2) receptor, mainly affecting the lower respiratory tract and its alveolar epithelia [5]. With respect to the clinical spectrum of the disease, it is reported to be wide. There may not be manifestations at all in the case of asymptomatic patients. When symptoms take place, they can appear in a mild, moderate, or severe way, with most of them being quite similar to the ones associated with colds or mild influenza: coughing, fever, chills, shortness of breath, and fatigue. Chilblains, ageusia, and anosmia have also been described. Nevertheless, when it comes to severe cases, respiratory and extra-respiratory complications often appear, including ARDS, acute cardiac and vascular complications, septic shock, multiple organ disfunction, and respiratory failure, frequently requiring ventilatory support [6]. These complications are related to a status of uncontrolled inflammation due to an unusually strong release of cytokines, which is triggered by viral replication. This response affects the lungs, resulting in pulmonary tissue damage, functional impairment, and reduced respiratory capacity [6]. As a matter of fact, two different phases of immune response have been reported during COVID-19. Firstly, a defense-based protective activity takes place, and afterwards, a second phase characterized by broad inflammation. Consequently, strategies for therapeutic management should try to enhance and boost immunity in the first phase and suppress it during the second one. In mild stages of the illness, the most advisable strategy would be stimulating the immune system. In contrast, once complications have already taken place, the preferable intervention should be oriented to immunosuppression [7].

Severe national policies have been set up to control the propagation of the disease, owing to the rapid evolution of the outbreaks, which have kept happening in several countries even when the pandemic seemed to be controlled. These mitigation strategies include the use of filtering facepieces, stay at home measures (e.g., lockdown or home working), social distancing, expansive/individual testing, and contact tracking. Even though some therapeutic alternatives look promising, there is a lack of solid evidence on the safety and efficacy of these proposed treatments in well-designed human trials. With regard to prophylactic interventions, the quick development and distribution of vaccines has been fundamental so as to control and mitigate the effects of the pandemic. However, the process of vaccination has not been fully completed, as it has not reached the whole population worldwide and recurrent doses seem to be necessary to avoid a poor prognosis of the disease [2].

For this reason, it has become necessary to improve the insight into other interventions that may help prevent the illness or improve its management. Within this context, there are some factors that could be considered, such as age, nutritional status, lifestyle features, or underlying conditions, in order to improve immunity before virus contact, and as a complement to vaccination or treatments. During infections, there is an augmented need for substrates, energy sources, and regulatory molecules, owing to the required enhancement of immune activity and the subsequent increased metabolism [2]. People usually neglect the optimal dietary patterns that provide a healthy nutritional status, which has been even more frequent during these stressful times of restrictive policies. However, an immune response that allows an optimal modulation of both oxidative stress and inflammation, clearly requires an adequate provision of several nutrients (sometimes even beyond the classic and established essential requirements), provided by a balanced diet and, if necessary, suitable supplementation [8]. Among the nutrients that should be taken into consideration, mineral supply must be highlighted, as they exert a wide range of mechanisms associated with immune function and oxidative stress that would certainly be beneficial during COVID-19. This is supported by the current knowledge in the scientific literature related to similar respiratory infectious diseases and, sometimes, SARS-CoV-2 infection itself. Thus, the following sections address the existing link between immunity, oxidative stress, and COVID-19 [9], together with a compilation of the scientific insights into the relationships between several minerals and this disease. The review focuses on the trace elements zinc, selenium, copper, and iron, and other minerals such as magnesium, as they exert the most relevant oxidative and immune-related effects, having been the most studied by the scientific community within the context of COVID-19 and other respiratory infections. However, other minerals with a minor scientific impact are addressed, as they have shown noteworthy mechanisms of action that should also be taken into consideration.

## 2. Materials and Methods

Bibliographical research was started in January 2022, and continued until the end of March 2022 when the current review was carried out. The period between 2019 and 2022 was chosen to focus the search on, making use of the main biomedical databases and sources: Medline (via PubMed), The Cochrane Library, Elsevier, and Dialnet. Acceptance was only considered for relevant articles that had been published in recent years, with all of them being related to the subject of study. Greater attention was paid to those articles associated with COVID-19 and the relation that minerals may have with it. The search was made just considering articles in English, as it is the lingua franca of the scientific field. As for the key words used to do so, these were: COVID-19, SARS-CoV-2, minerals, trace elements, zinc, selenium, copper, iron, magnesium, nutrition, nutrients, inflammation, and oxidative stress. Medical subject heading (MSH) terms were applied in the words that might create a misunderstanding in the browser. In addition, the applied Boolean operators were “AND”, “OR”, and “NOT”, which were combined with key words in order to find useful and valuable articles. “AND” was used between every word to provide more sensitivity and specificity to the search. “OR” was applied to connect synonyms. “NOT” was not frequently used to avoid confusion in the browser.

**Figure 1:**
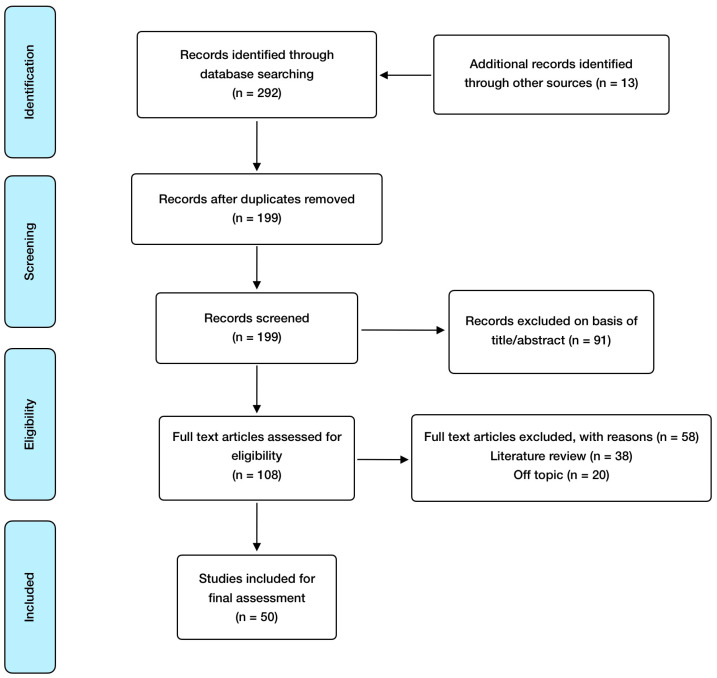
Manuscript selection flowchart.

Regarding inclusion criteria, these included: randomized controlled studies, observational studies, meta-analysis, animal model and in vitro studies; published in English and open access, with special consideration for studies involving humans or animals with a SARS-CoV-2 infection clinical diagnosis. Exclusion criteria included: absence of abstract and publication in a language different from English. EndNote was used as a reference software for article management and bibliography organization. In relation to search methodology, Figure 1 shows the selection process carried out, and Table 1 summarizes the articles finally included and reviewed, referring to the addressed mineral, the study design, and its major findings.

## 3. Results and Discussion

### 3.1. The Link between Immunity, Oxidative Stress, and COVID-19

Microbe activity is not the only determinant that influences an infectious disease’s development and outcome. Immunity and oxidative stress also have a crucial relevance, being able to modify the consequences of the infection [60]. In order to obtain entry to the organism, pathogens need to overcome natural barriers that protect the body against them, so an adequate membrane state and junction integrity between cells is undeniably necessary to prevent microbes from invading tissues. The implication of mucosal IgA in the protection of these physical barriers against viruses has been well documented, to the extent that an increase in this immunoglobulin’s response has been reported in severe cases of SARS-CoV-2 infection [61].

After microorganisms get through this first line of defense, they must face the two compounds of immunity, innate and adaptive responses, which try to neutralize and eliminate them. Even though the exact interactions between the immune system and the novel coronavirus have not been fully established yet, the functioning of immune cells appears to have a completely relevant role in the symptoms and severity of COVID-19. Previous studies carried out with its closest relative, SARS-CoV, support this assumption [62]. Once pathogen-associated molecular patterns (PAMPs) are recognized by pattern-recognition receptors (PRRs), this triggers signaling pathways that leads to the release of inflammatory mediators [63]. Coronaviruses encode several proteins that can interfere not only with this recognition process but also with the following activation of viral controlling mechanisms. In particular, they can block viral RNA recognition and subsequent IFN responses thanks to viral M protease action [64].

Cytokine release acts as a primary inflammatory response against SARS-CoV-2, especially interleukin-6 (IL-6), interleukin 1β (IL-1β), and tumor necrosis factor alpha (TNFα), which stimulate its receptors and activate intracellular signaling pathways, including nuclear factor kappa-B (NF-κB) [65]. A certain level of acute inflammation is physiologically necessary to obtain an optimal immune response after the exposure to a certain pathogen. However, low-grade chronic systemic inflammation is associated with disturbances and alterations in the immune system, and consequently, a bigger risk of infection and a poor prognosis. This type of inflammation is a frequent feature in several diseases, including type 2 diabetes (T2D), cardiovascular disease (CVD), overweight/obesity, metabolic syndrome, arthritis, and cancer [66], which are all considered to be comorbidities for those patients suffering from COVID-19. In this sense, extreme inflammatory conditions delivered by a severe SARS-CoV-2 infection might be life-threatening for patients, particularly if they also suffer from any of these conditions.

As a matter of fact, the main cause of complications related to coronaviruses-derived severe pneumonia is an excessive inflammatory response following viral replication [67]. An augmented release of cytokines, chemokines, and other immune molecules has been reported during these infections, including IL-1β, IL-4, TNFα, interferon gamma (IFN-γ), interferon inducible protein 10 (IP-10), IL-2, IL-7, monocyte chemoattractant protein 1 (MCP-1), and granulocyte colony-stimulating factor (G-CSF), especially in hospitalized patients from intensive care units (ICU). In addition, bronchial epithelial cells’ reaction to SARS-CoV-2 and SARS-CoV has also been reported, owing to an increased release of IL-6 and IL-8, which are NF-κB-mediated cytokines [67]. Detrimental consequences in patients’ recovery have been related to high IL-6 production, which might explain the augmented serum levels of C reactive protein (CRP) observed in these cases, something frequently lacking in infections caused by viruses [62]. As a result, this situation, also called “cytokine-storm”, may result in hyperinflammation, immune cells’ infiltration, and edema, leading to serious complications, including immune exhaustion, hyperpyrexia, ARDS, thrombosis, and even multiple organ failure [67]. A severe cytokine profile in COVID-19 patients has undeniably been correlated with poor therapeutic outcomes [68]. As a conclusion, research findings highlight the fact that inflammatory responses during COVID-19 are more harmful and life-threatening than the virus’s own activity [62].

Regarding lymphocytes, some peculiarities have been observed in SARS-CoV-2 infections. For instance, it has been reported that there is an increased exhaustion and a decrease in natural killer (NK) cells’ and cytotoxic CD8+ cells’ activity, particularly in the early stages of the disease, which leads to a more severe progression. A lower count of these cells has also been observed, and in some cases, the trend is shared by CD4+ cells and regulatory T cells. An association between the augmented pro-inflammatory cytokines profile and the depletion/functional distress of T lymphocytes has also been reported [69,70]. As for antigen presentation, SARS-CoV-2 can restrain this process by down-regulating the major histocompatibility complex (MHC), which impairs immune responses mediated by T cells, just like other coronaviruses are able to do. On the other hand, humoral immunity also has an important role in the disease, helping neutralize this new virus. After the infection has been set up, B lymphocytes generate antibodies that can prevent SARS-CoV-2 from obtaining access to host cells, also playing a critical part when it comes to virus clearance [69]. Furthermore, studies have described the spleen and lymph nodes of COVID-19 patients as atrophic, something that highlights the role of this virus in immune-related cell damage and degeneration [70]. All told, these findings point out that SARS-CoV-2 exerts an immunosuppressive effect on immunity, especially on its adaptive component. Many of the trace elements and minerals addressed in this review carry out mechanisms that would come in handy to reduce cytokine storm severity and enhance immune cells’ activity.

As for oxidative stress, its role in this disease should also not be underestimated. The situation is characterized by the presence of an imbalance between the amount of reactive oxygen species (ROS), reactive nitrogen species (RNS), reactive sulfur species (RSS), and the activity carried out by different antioxidant systems: reduced glutathione, some vitamins (especially C and E), and endogenous enzymatic systems such as superoxide dismutase (SOD), catalase (CAT), or glutathione peroxidase (PXD) [65]. It is important to highlight that some trace elements frequently act as cofactors of several of these crucial enzymes. An excess of extracellular free radicals is rather detrimental, as they can oxidize important bio-macromolecules, including membrane lipids, proteins, RNA, and DNA, with them being able to change the structure of both proteins and genes related to inflammatory signaling pathways [71]. As a result, there is a tight relationship between oxidative stress and inflammation, which has been widely documented. Free radicals generated in the site of infection by different cells of the immune system, especially macrophages and neutrophils, have the aim of neutralizing and killing invading microbes [72]. Nevertheless, when an infection progresses in a long-term way, oxidative stress becomes chronic, with it being associated with altered immune responses, a higher level of inflammation, and endothelial damage. All of these are situations that play a pivotal role in COVID-19 development and prognosis [73,74].

The affectation of lung histology and function after COVID-19 is rather severe. Diffuse alveolar damage (DAD) is the most frequent finding in patients’ histopathology. DAD is a lung manifestation characterized by the presence of interstitial edema, hyaline membranes, and fibroblast proliferation. Apart from these, other pathological features of DAD are enlarged and atypical type II pneumocytes, squamous metaplasia, and the appearance of thrombi in small pulmonary arteries (due to infection-related endothelial damage). Less frequently reported pathological findings for the disease are intra-alveolar fibrin, endotheliitis, and viral inclusions [75].

The immune system is interrelated with a variety of physiological features, such as circadian rhythms, hormonal/metabolic regulation, and nutrition. In general, a state of malnutrition can strongly compromise immunity, as it might affect immune cells’ proliferation and functioning, making patients increasingly prone to infectious diseases as a result [76]. In addition, the level that inflammation associated with unhealthy dietary habits is reaching nowadays is rather alarming, and this has an undeniable connection with the coexistence of several non-communicable diseases (NCDs). These sicknesses may aggravate the inflammatory and oxidative pathology of SARS-CoV-2 infection, and even augment the likelihood of complications and mortality [77]. This chronic low grade of inflammation can be counteracted through dietary and nutritional strategies, such as the fulfillment of the advised nutrient daily intakes through food or supplementation. This would decrease the risk of infection, including from COVID-19, and consequently reduce the likelihood of a fatal outcome [65]. An increasing body of evidence has supported the aforementioned fact, which suggests that this intervention might be one of the best choices in order to strengthen the immune system and avoid an excess of inflammation and oxidative stress. A proper immune response prevents progression to severe stages of the infection, so following strategies oriented to enhance immunity could be determinant in the early stages. In this sense, several nutrients might be useful in COVID-19 management as an adjuvant therapy, with them being involved in additional pathways such as the interaction with relevant proteins of the viral life cycle [7].

The following sections review the roles that several minerals (focusing on zinc, selenium, copper, iron, and magnesium, but also including some other relevant ones) have in immunity, inflammation, and oxidative stress. Table 2 summarizes the most relevant clinical trials concerning mineral supplementation and COVID-19, including doses, population of study, and major findings. Likewise, these sections establish their importance for preventing the infection during these times, ameliorating the development of the disease that has already taken place, and promoting healthy nutritional habits.

### 3.2. The Role of Zinc (Zn)

Zinc is the most abundant trace metal in humans after iron, being crucial for a variety of cellular functions as it serves as a cofactor, structural element, and signaling molecule [78]. Its proteome includes about 3000 proteins, highlighting 750 zinc-finger transcription factors in whose structure the trace element is located [79]. These proteins are involved in DNA and RNA synthesis, thus being necessary for protein generation and many homeostatic functions [65]. However, Zn acts as a cofactor for many other enzymes, some of them involved in host antioxidant defense, such as superoxide dismutase (SOD). In this sense, a suitable status of this mineral would be essential for a proper cell protection against free radicals [80].

The role of zinc in the physiology of the immune system is well known, mostly due to its activity as a signaling molecule. It is necessary for the differentiation and growth of immune cells, allowing a rapid replacement rate [78]. This is crucial for adaptive immunity, and so it is a fact that Zn acts as a second messenger in the signal cascade after the activation of T cell receptors by IL-2, allowing the proliferation of these cells [81]. Zinc plays an important role in the thymus gland, being an essential component of thymulin, a hormone involved in the maturation of T lymphocytes. The element also has a relevant effect on bone marrow, and its deficiency leads to a decrease in the number of immune precursor cells. Therefore, this deficiency would be translated into a limited output of naive lymphocytes (both T and B), together with thymus atrophy, lymphoid tissue atrophy, and lower antibody production. In addition, an imbalance among the different types of T cells appears, in favor of Th2 cells. Finally, the proliferation and activation of CD8+ T lymphocytes are also reduced, and consequently, their cytotoxic activity cannot fully collaborate in antiviral defense [82]. Lymphopenia is frequently associated with a poor prognosis of SARS-CoV-2 infection, having been described in rodents with zinc deficiency, even though this immunodeficient situation is reversible through supplementation [10].

As for innate immunity, zinc deficiency interferes with many aspects of it, especially phagocytosis, degranulation, cytokine expression, and respiratory burst, just as with complement activity. NK cells’ cytotoxic activity is also affected by low levels of this trace element [11,80]. Severe COVID-19 patients show a marked neutrophilia, which studies have demonstrated can be partially counteracted with zinc gluconate. This intervention decreased neutrophil infiltration into lung tissue, also reducing TNF-α release through the blockage of NF-kB-dependent inflammatory genes [83]. Studies also suggest that neutrophil recruitment and the formation of neutrophil extracellular traps (NETosis) are reduced owing to zinc supplementation [84]. NETosis is a cell death pathway, different from necrosis and apoptosis, whose main objective is pathogen inactivation. Nevertheless, an excess of this response is considered a maladaptive situation that contributes to tissue damage, thrombotic complications, and organ failure in several diseases, including COVID-19 [85]. Toll-like receptors (TLR) are another important host defense mechanism regarding innate immunity. In silico data show the potential for TLR-4 to recognize outer SARS-CoV-2 components (such as spike protein), and for TLR-3 to recognize viral RNA. TLR-3/4-induced signaling is regulated by zinc, so low levels of this element would disrupt the innate immune response against SARS-CoV-2, making it easier for the virus to enter and replicate within hosts [86]. Moreover, the Zn finger CCHC-type containing 3 (ZCCHC3) can induce TLR-3 signaling, which occurs during intracellular replication of RNA viruses such as coronaviruses [79].

Zn deficiency significantly alters cytokine production, with induced IL-6 and IL-1β expression, as well as intercellular adhesion molecule 1 (ICAM-1), which is necessary for the extravasation of leukocytes. In addition, this trace element acts as a cofactor of ADAM enzymes (a disintegrin and metalloproteinase) with a major role in inflammation, as they catalyze the activation of TNFα and the conversion of membrane-bound IL-6 to soluble IL-6 [11]. Therefore, Zn supplementation might be useful to reduce patients’ inflammatory profile caused by these cytokines. On the other hand, zinc also stimulates IL-12 expression by macrophages. The immune role of this cytokine is the promotion of NK cells and T cytotoxic cells’ activation, which is crucial in the destruction of several pathogens, especially viruses. With regard to anti-inflammatory cytokines, IL-10 is the only one affected by Zn deficiency, whose increased production softens Th1 cells’ and macrophages’ responses [87].

Interferon has a critical role in the host’s defense against viruses. However, it has been reported that SARS-CoV-2 is able to reduce IFN secretion, thus attenuating its antiviral effects and making it easier for the virus to develop and replicate during the disease. Zinc could be useful in order to avoid this negative feature of COVID-19, as studies developed in rhinovirus-infected cells have shown that supplementation is able to reconstitute IFN expression, possibly counteracting the antagonism against this cytokine exhibited by SARS-CoV-2 and restoring this defensive immune response [88]. IFN is a crucial cytokine for Th1 cells’ development, also being responsible for the inhibition of Th2 cells. The down-regulation of IFN expression induced by Zn deficiency could also lead to an imbalance between these two types of cells, causing a dysfunction in cell-mediated immunity [79]. In addition, ACE2 expression could be dependent on IFN secretion, so the effects of zinc on this subject should be more carefully evaluated to fully elucidate its potential [89]. In summary, optimal zinc levels would help maintain an optimal immune response, whereas deficiency would be associated with a defective response and an increased susceptibility to a number of infectious diseases, including pneumonia [90]. This can be observed, for instance, in acrodermatitis enteropathica, a Zn malabsorption syndrome whose patients exhibit serious immune impairments. However, this faulty response has been proven to be corrected by zinc supplementation and repletion [82].

Notably, the role of zinc in antiviral immunity has also been highlighted, as its unchelated free form and some ionophores (such as pyrithione) have been associated with direct antiviral effects against a great amount of pathogens, including coronavirus, the influenza virus, picornavirus, rhinovirus, herpes simplex virus, respiratory syncytial virus, hepatitis C virus (HCV), and human immunodeficiency virus (HIV) [78,91]. The antiviral roles of zinc include a reduction in viral polymerase activity, the prevention of viral fusion with the host cell membrane, the disruption of protein translation and processing, the destabilization of the viral envelope, and the blockage of viral release. Zinc acts as a cofactor for a variety of viral proteins, but it can also serve as a second messenger able to trigger apoptosis of infected cells. One of the most relevant mechanisms is related to RNA polymerase inhibition, especially in RNA viruses such as rhinovirus and coronavirus, as they depend on it to replicate [91]. Influenza virus replication has also been attenuated by Zn in some in vitro studies, and something similar happened with SARS-CoV-1 when the trace element was administered with pyrithione. These facts strengthen the possibility that SARS-CoV-2 replication may also be affected by it [12]. Furthermore, zinc-binding metallothioneins seem to be involved in antiviral defense too, as evidence points out that their expression is augmented during responses against infections [92].

The relationship between zinc and host physical barriers is also well documented, having a significant role in the prevention of viral entry. Firstly, zinc affects mucociliar clearance of microorganisms, as it can amplify ciliary beat frequency when it is present at physiological levels. In addition, it is capable of increasing the length and number of bronchial cilia. Therefore, this trace element has a positive effect not only on the removal of viral particles, but also for decreasing the risk of subsequent bacterial infections [93]. On the other hand, zinc is also essential to preserve tissue integrity, which is vital when it comes down to coronaviruses and respiratory epithelia. Studies have demonstrated that reduced Zn levels augment leakage in the respiratory tract and produce changes in the extracellular matrix, whereas supplementation improves lung tissue integrity. Zinc also raises the expression of tight junction proteins, which together with its ability to increase lung tolerance towards mechanical ventilation-induced damage, offers different explanations for zinc’s beneficial effects on lung tissue [10,13].

**Figure 2:**
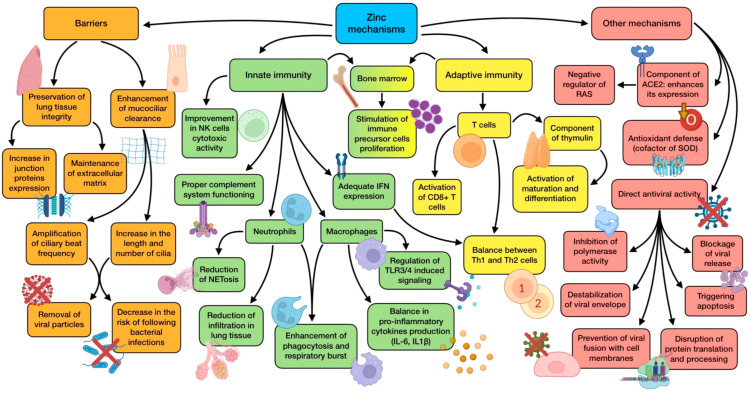
Summary of zinc’s main mechanisms of action (references detailed in brackets). Through them, this trace element exerts several effects related to physical barrier enhancement [10,13,93], innate immunity [11,79,80,83,84,85,86,87], adaptive immunity [10,78,81,82], as well as other mechanisms associated with ACE2 expression [85,89], antioxidant defense [80], and direct antiviral activity [12,78,91,92]. Color description: orange (barrier enhancement), green (innate immunity), yellow (adaptive immunity), and red (other mechanisms).

Another relevant mechanism is related to ACE2. This is a zinc metalloenzyme in whose active center the element is located, being crucial for its enzymatic activity and possibly for the stability of its structure. Zinc may affect ACE2 expression, as something similar has been reported for other zinc metalloenzymes, including matrix metalloproteinases and metallothionein [89]. Positive effects of ACE2 expression are associated with its ability to inactivate angiotensin II, thus acting as a negative regulator of the renin–angiotensin system (RAS). As a result, it has an important role in regulating vascular permeability, coagulation, inflammation, and their negative pulmonary consequences during ARDS development. This mechanism somehow clashes with the fact that SARS-CoV-2 needs ACE2 to get inside the cells. However, the binding of S1 spike protein to ACE2 causes the translocation into the cell of both the virus and the protein, decreasing its expression and contributing to the development of a more severe pulmonary pathology [85]. A summary of zinc’s mechanisms of action is shown in Figure 2.

Zinc deficiency, far from being an uncommon issue, is a prevalent situation worldwide, even in Westernized countries [65]. The WHO estimates that at least one-third of the population is affected by this problem, which frequently stays subclinical and unnoticed when the severity of the deficiency is rather mild. Aged and infant populations are particularly susceptible to suffering from it [94]. The wide range of mechanisms and immune components affected by a lack of zinc makes this situation a big contributor to the increased risk of infections, especially those that affect the low respiratory tract, the skin, and the gastrointestinal tract. Moreover, information also provided by the WHO points out that zinc deficiency is related to 16% of deep respiratory infections. In this sense, the correction of this deficiency would possibly decrease the likelihood and severity of these diseases, possibly having a potential benefit in the context of the COVID-19 pandemic [80].

The available Information about the role of zinc in SARS-CoV-2 infection is limited, so most of the current knowledge about this topic is based on studies performed in different but similar diseases. Zn is considered a popular remedy against the common cold. It has been suggested that it is able to ameliorate its symptoms, with a randomized double-blind study demonstrating this premise. A dose of zinc was administered to patients with cold symptoms so long as they were present, and consequently, the duration of these was reduced from 7.6 to 4.4 days, compared to a placebo [95]. Systematic reviews and meta-analysis also point out a reduction in not only the frequency but also the duration of colds by around a day [14,15]. It is important to recall that 30% of common colds are produced by coronaviruses [89]. In addition, studies performed in Chinese children reported that the ones prone to recurrent upper respiratory tract infections (URTI) had an increased likelihood of having low hair Zn [16]. As for the elderly population, low zinc status has been related with pneumonia, with a mortality rate twice as high in people with deficiency versus individuals with a proper status of this trace element [96]. Studies on zinc supplementation also reported a reduced incidence of pneumonia in children and a lower mortality rate in adults affected with serious cases of the disease [82]. In most studies carried out about zinc and respiratory tract infections, prophylactic supplementation proved to be more efficient than therapeutic use.

According to all these findings, the suggestion that a proper Zn intake or appropriate supplementation would be useful against SARS-CoV-2 infection is gaining strength, with the possibility of reducing viral replication and attenuating the impact of respiratory and gastrointestinal symptoms [90]. In vitro approaches have highlighted an inhibition in SARS-CoV replication produced by zinc through a blockage of polymerase activity [12]. Consequently, optimal Zn status should be considered as a strategy to reduce COVID-19 effects. In this sense, vulnerable populations such as pregnant women have shown lower serum zinc levels and Zn/Cu ratios during the COVID-19 disease, with these parameters being negatively associated with acute phase markers such as IL-6 and CRP [17]. Recent studies also remark on the direct relationship between the disease and this nutrient, highlighting that maintaining its status within the reference ranges augments the survival odds in COVID-19 [18]. In consequence, zinc treatments are being tested in clinical trials (both alone and as an adjuvant) on COVID-19 [97]. Among these, a recent study highlighted that oral zinc can decrease the ICU admission rate, hospital stay, and 30-day death, and also shorten symptom durations [19]. The administration of a food supplement (containing zinc, selenium, vitamin D, probiotics, and prebiotics) proved to be a protective factor in severe COVID-19 patients, which shortened the hospital stay and allowed early recovery from digestive symptoms [20]. Moreover, 7-day supplementation of zinc and ascorbic acid in SARS-CoV-2 infections without clinical symptoms significantly reduced the viral load, with none of the cases progressing to a symptomatic state [21]. In a medical report, four COVID-19 patients were treated with oral high doses of zinc, exhibiting a noticeable improvement after one day, characterized by a significant reduction in symptoms [22]. A subsequent study verified the safety of similar interventions with high doses of intravenous zinc, which was reported to be safe and feasible. However, no outcomes or conclusions about the recovery or ventilation need could be drawn due to a lack of enrollment [23].

The trace element has also proved to be useful in ameliorating some of the most characteristic clinical symptoms of COVID-19 such as olfactory and gustatory disturbances. Zinc treatment shortened the mean duration of these alterations, while the duration of complete recovery from the disease was not significantly different compared to a placebo [24]. Similar Zn interventions have also been carried out to study its prophylactic role, especially for healthcare workers. In this sense, a significant decrease in SARS-CoV-2 infection in this population was reported when they received both doxycycline and zinc, instead of just the mentioned drug [25]. On the other hand, the efficacy of zinc has been tested when it comes to improving the COVID-19 vaccine response. Levels of CD4+T lymphocytes increased after the administration of a synergistic combination of β-glucans with selenium- and zinc-enriched *Saccharomyces cerevisiae*, with changes in CD8+T and CD3+T cells showing a similar trend. Augmented IgG and IgM were also reported [26]. All this information suggests that adjuvant therapy with zinc might have a relevant role in clinical recovery, associated with immunomodulation, the reduction in lung inflammation, the enhancement of mucociliary clearance, and reduction in ventilator-induced lung injury [79]. Nevertheless, not all the studies carried out reached positive results. Some of them did not find a significant decrease in the duration of symptoms after treatment with high-dose zinc gluconate, ascorbic acid, or a combination of both, compared with standard care [27]. Likewise, supplements of this trace element did not improve the clinical efficacy of drugs such as hydroxychloroquine [28].

Broadly speaking, Zn levels are significantly reduced during infection, and its requirements seem to increase together with the severity of the disease. This fact supports the use of zinc supplementation to restore its balance in the organism. However, although its use as a supplement or adjuvant is usually safe, it can become a two-edged sword if used improperly. Excessive doses administered over a long period of time can modify immunity, altering neutrophil and lymphocyte functioning, and finally leading to a suppression of the immune system [79]. An excess of zinc can also be negative for a proper antiviral response, as it can ironically decrease the production of IFN-γ. Large doses might also lead to copper deficiency since zinc might block and hinder its intestinal absorption. As a consequence, Zn levels should be studied and monitored so as to obtain the maximum efficacy from this intervention [29].

Most of the research concerning zinc and infections has been focused on supplementation. Therefore, there is no solid evidence that high dietary zinc can protect against viruses and bacteria by itself. Nevertheless, the importance of meeting the nutritional requirements of this nutrient is not in doubt, always taking care of the influence of phytate, an antinutrient capable of hindering its absorption [98]. In conclusion, and based on the available knowledge about its potential benefits and risks, zinc supplementation in COVID-19 patients seems to be favorable. However, clinical data specifically about SARS-CoV-2 infection are still limited, so future results from current clinical trials could help elucidate the actual potential of this trace element.

### 3.3. The Role of Selenium (Se)

Selenium is required for the synthesis of several enzymes called selenoproteins. They all have in common the presence of a selenocysteine amino acid residue in their sequence, especially at their active center. These groups of proteins include glutathione peroxidases (GPX), thioredoxin reductases (TXNRD), methionine sulfoxide reductase B1 (MSRB1), and selenoproteins (P, K, W, S, etc.) [99]. They exert a wide range of relevant functions in case of infection:

- Antioxidant activity: the majority of selenoproteins have a role in the mitigation of oxidative stress, being able to neutralize free radicals triggered by infections, immune response, and inflammation [99].
- Maintenance of cellular redox homeostasis: TXNRDs and GPXs are required for preserving mitochondrial integrity and redox tone in immune cells, reversing oxidative damage inflicted on them (in part due to its own oxidative burst) [100].
- Anti-inflammatory effects: selenoproteins take part in the generation of anti-inflammatory mediators derived from arachidonic acid (AA), which protect cells against the expression of pro-inflammatory genes. They can also inhibit NF-κB, thus down-regulating inflammatory genes. In addition, these proteins activate the peroxisome proliferator-activated nuclear receptor-γ (PPAR-γ), which leads to a repression of pro-inflammatory genes [101,102].
- Immunomodulatory effects: these types of proteins can modify immunity through an up-regulation of IL-2 receptor’s expression. Therefore, T and B cells can respond easily to this cytokine, increasing their proliferation and activation. Moreover, selenoproteins P, W, and K modify circulating levels of some cytokines, such as IL-6, TNF-α, and IL-1β [102].
- Other functions: TXNRD1 is necessary for DNA synthesis during T cell proliferation. GPX1 activity is related to a reduction in cardiovascular events in patients suffering from coronary disease, and TXNRD2 has been proven to preserve heart functioning in the elderly [103].

There is already evidence provided by in vitro studies that point out a negative effect of SARS-CoV-2 in selenoproteins’ expression, as infected cells experiment with the reduced production of many of them [30]. Selenoprotein S is one of the proteins whose expression is down-regulated during this infection, with a well-documented relation between its reduced production and the release of pro-inflammatory cytokines. This suggests that selenoproteins may be a possible link between COVID-19 and increased IL-6 levels detected during the disease [102].

The association between selenium and the immune system is way deeper, as this element is involved in both acquired and innate immunity, improving the differentiation and normal functioning of a number of its components. It promotes the proliferation of T cells and enhances humoral immunity through an increase in immunoglobulin production by B cells. Supplementation has also been proven to be involved in ameliorating cytotoxic T cells’ and NK cells’ activity by up-regulating the expression of IL-2 receptors, which is crucial for proliferation and growth regulation in these cells [65]. Suboptimal levels of selenium correlate with reduced albumin concentration (which is a marker of protein status) and with augmented levels of CRP [31]. This situation is also associated with impairment in the cellular immune response and reduced antibody titers, which is translated into a faulty response to vaccination. Consequently, deficiency brings an increased susceptibility to infections, especially the viral ones [98]. Furthermore, selenium deficiency seems to favor an imbalance between Th1 and Th2 responses, with an excessive activity provided by Th2 lymphocytes. This situation can be reversed through Se supplements, leading Th1 to a preferential position. Th1 predominance can be useful when it comes to vaccination and the early stages of the disease, as it has been shown to be related to a more robust response, for instance, in polio immunization [100]. Nevertheless, this effect of selenium in the Th1/Th2 ratio and its consequences needs further investigation, as the Th1 phenotype is associated with stronger cellular immunity but, at the same time, can raise the release of some inflammatory cytokines, which would cause more damage to severe COVID-19 patients [102].

Macrophages are another component of immunity (innate in this case) that are also affected by selenium supplementation. In particular, the trace element makes these cells attenuate their pro-inflammatory reaction, characterized by cytokine release. Additionally, Se deficiency has been reported to directly inhibit macrophage phagocytosis [104]. IL-6 is one of the main cytokines released by active macrophages, and its implications in the COVID-19 cytokine storm is well known, being an interesting target in order to decrease this exaggerated response. Selenium could be of use in this subject, as the available knowledge highlights the ability of this trace element to down-regulate IL-6 production. Its deficiency has also been associated with higher concentrations of the cytokine, especially in older populations, supporting an inverse correlation between IL-6 and Se. Therefore, a proper selenium status seems to contribute to optimal IL-6 levels, both in patients and healthy subjects [32]. Transcription factor NF-κB coordinates the activation of pro-inflammatory cytokines, being a key element in the inflammatory process. In this sense, cell culture studies have shown that selenite may act as a specific inhibitor of the factor. In addition, uncontrolled ROS production promotes NF-κB activation, so Se and selenoproteins such as GPX would also reduce its activity by reducing the oxidative stress that triggers its functioning [102]. Another fact that supports this anti-inflammatory role of selenium is Kashin–Beck disease, a condition related to selenium deficiency, and whose patients show increased levels of inflammatory cytokines, including not only IL-6 but also IL-1β and TNF-α [105].

All these mechanisms support the potential role of selenium adjuvant therapy in viral infections to fight against them and ameliorate the state of patients. Positive results in viral inhibition have been found for the influenza virus, coxsackievirus, hepatitis C virus (HCV), and poliovirus [106]. Supplementation has also been demonstrated to have protective activity against cytomegalovirus (CMV) and its heart-damaging effects [65]. In addition, poor Se status seems to be related to a higher mutation rate in viruses, which could lead to the appearance of more pathogenic strains that would increase the risk and worsen the outcomes of infections. This has been observed in selenium-deficient models with several viruses, including coxsackievirus, murine influenza virus, and poliovirus [107].

Another relevant fact concerning selenium and viruses is that some of them exert mechanisms that could alter the selenoproteome. One of these mechanisms is related to virally encoded glutathione peroxidases (vGPX), which would directly compete with host cells for selenocysteine, making its presence quite limited within infected cells. These viral proteins have been demonstrated to be encoded in the genetic material of both RNA and DNA viruses [108]. Another mechanism is the degradation of host selenoproteins through proteolysis performed by viral proteases. The existence of a strong interaction between GPX1 and a mutant of M-pro (one of SARS-CoV-2 main proteases) has recently been demonstrated, a discovery supported by the finding of potential M-pro cleavage site sequences in GPX1, TXNRD1, and selenoprotein F. These attacks developed by several viruses against selenoproteins, also at an mRNA level, would strengthen the role of selenium status in the pathogenesis of infections, including COVID-19 [33,109]. However, regarding COVID-19, the influence between the virus and Se is not limited to the degradation of selenoproteins by M-pro. This relation is bidirectional, as selenium has also been proven to be a potent inhibitor of viral proteases. SARS-CoV-2 replication requires a proteolytic processing of the two polyproteins encoded by the viral genome. This process is mainly carried out by M-pro, though there is another protease called papain-like protease (PL-pro) that is also pivotal for the viral life cycle. As these proteins allow the formation of mature and functional viral subunits, they have become an attractive target in the design of antiviral drugs. Based on this, a screening of 10,000 compounds has been performed, including approved drugs, drug candidates under clinical/preclinical trial, and natural products, in order to find possible inhibitors. Among them, an organoselenium compound called “ebselen” reported the strongest inhibitory activity, owing to its ability to covalently bind to a cysteine residue located in the active site of the M-pro. Additionally, ebselen performed as an important suppressor of PL-pro through another covalent binding to a cysteine residue in the active center [34].

**Figure 3:**
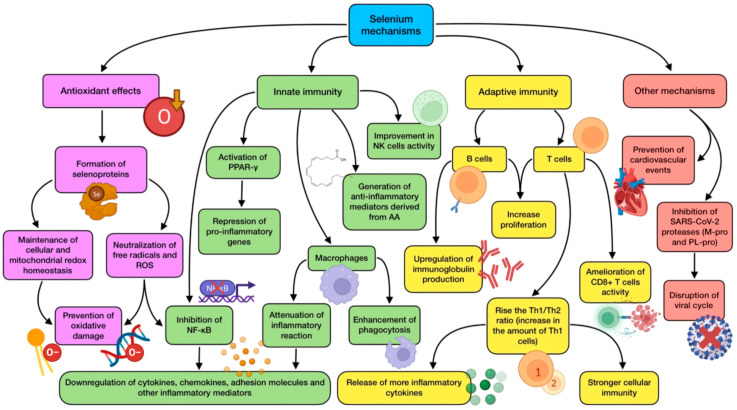
Summary of selenium’s main mechanisms of action (references detailed in brackets). Through them, this trace element exerts several effects related to antioxidant defense [99,100], innate immunity [32,65,98,101,102,105], adaptive immunity [100,102,103], as well as other mechanisms associated with cardiovascular protection [103], and SARS-CoV-2 viral cycle disruption [33,34,109,110]. Color description: purple (antioxidant defense), green (innate immunity), yellow (adaptive immunity), and red (other mechanisms).

The effects of a high selenium intake in the diet or by supra-nutritional supplementation have been described. When Se levels are beyond the amount needed for selenoprotein biosynthesis, selenium compounds with small molecular weight (methylselenol, dimethylselenide, and hydrogen selenide) accumulate in cells. They are all volatile molecules, thus likely appearing in the respiratory tract. When cells contain high concentrations of oxidants, methylselenol remains a non-volatile compound after being transformed into methylselenilic acid. As viral respiratory infections such as COVID-19 are known for inducing ROS generation, methylselenilic acid would accumulate in infected cells, having the ability of inactivating M-pro protease through a modification of its Cys145 residue [102,110]. The main mechanisms of action exerted by selenium are summarized in Figure 3.

Considering all the mechanisms mentioned above, it is not surprising that studies concerning selenium supplementation have shown an improvement in the development of respiratory diseases in humans. The elderly seem to benefit from this improvement too, which is important since they are the population group with a greater risk of a serious outcome. Supplementation in individuals with low Se status also improves their immune response to vaccination [111]. Selenium deficiency has been reported in multiple populations worldwide, including in Westernized countries [112]. It has been associated with an increased number of respiratory infections such as influenza. Levels of Se have been studied in patients with respiratory diseases treated in ICUs, concluding that serum concentrations at admission were 28% lower in these patients than in control subjects [31]. A recent randomized clinical trial provided sodium selenite to critically ill patients suffering from ARDS. This selenium supplementation proved to restore the lungs’ antioxidant capacity, ameliorate breathing, and modulate the inflammatory response. The levels of this compound showed a linear association with GPX presence and antioxidant activity, and an inverse association with IL-6 and IL-1β concentrations. Nevertheless, these findings did not translate to positive significant effects on survival, ICU stay, or need/duration of mechanical ventilation [35].

With regard to SARS-CoV-2 infection, recent data suggest that proper levels of selenium may support immunity against the disease. Firstly, a great example of this element’s relevance to COVID-19 is the analysis of the disease cumulative data in specific Chinese cities [36]. Enshi city, located in Hubei province (where the first cases appeared), is renowned for the high Se intake of its citizens. Noticeably, the cure rate (36.4%) in this place was significantly higher than in other Hubei cities (13.1%). Furthermore, data from Heilongjiang province, whose serum selenium levels are especially low, showed a higher death rate (2.4%) compared to other provinces (0.5%). In conclusion, a positive correlation between selenium status (determined by measurement of hair concentration) and recovery rate in COVID-19 was found. In addition, countries with the highest case fatality rates regarding COVID-19 (such as Italy, United Kingdom, Spain, or France) are also those with a higher previously documented deficient selenium status. Countries where Se intake and levels are adequate seem not to be that affected by the virus, showing less worrisome indicators related to the pandemic [112]. Reinforcing this association, a study found a significant deficit of serum Se and selenoprotein P levels in patients infected with SARS-CoV-2, which supports the use of Se supplementation as a way to restore the concentrations of these protective factors [37]. The testing of selenium in clinical trials has been scarce, and always combined with other compounds and nutrients. For instance, the administration of a food supplement (containing Se, Zn, vitamin D, probiotics, and prebiotics) proved to be a protective factor in severe COVID-19 patients, which shortened hospital stay and allowed early recovery from digestive symptoms [20]. Selenium has also been shown to be beneficial in the prevention of COVID-19-derived complications, with sodium selenite being proposed as a therapy to decrease the likelihood of blood clot formation [98]. Furthermore, the efficacy of this trace element has been tested when it comes to improving the COVID-19 vaccine response. Levels of CD4+T lymphocytes increased after the administration of a synergistic combination of β-glucans with Se- and Zn-enriched *Saccharomyces cerevisiae*, with changes in CD8+T and CD3+T cells showing a similar trend. Augmented IgG and IgM were also reported [26].

Among the different risk factors for a COVID-19 fatal outcome, a lot of them also have a negative influence on selenium status, including age, obesity, and chronic obstructive pulmonary disorder (COPD). Therefore, patients with conditions such as these would possibly benefit the most from supplementation, even though low Se levels might be a result of the associated inflammatory state, and factors different from Se may also be relevant [113]. Nonetheless, not everything is positive when it comes to selenium supplementation. It can be a double-edged sword due to its low therapeutic width. Excessive levels of Se owing to supplements have been associated with an increase in the incidence of type 2 diabetes [65]. The current literature does not recommend an increase in selenium intake for individuals with adequate levels of this trace element, as they can possibly reach levels normally related to toxicity produced by redox-active selenium species. However, the lack of adverse effects resulting from supra-nutritional doses of Se in critical patients supports the idea that toxicity would be rather unlikely in COVID-19, possibly providing a benefit to those with moderate to severe symptoms [114].

In the light of the evidence mentioned above, it can be assumed that ensuring a proper selenium intake and optimal status could be a strategy for boosting the immune response, attenuating oxidative stress and excessive inflammation, and preventing viral infections, among which COVID-19 can be included. Promising results have arisen from studies on supplementation in critically ill patients, though further analyses are required, especially on SARS-CoV-2 infection, to elucidate if supplementation could confer a survival benefit. However, precautions need to be taken when using supplementation of selenium salts in order to prevent possible negative effects on patients’ health [102].

### 3.4. The Role of Copper (Cu)

Copper is an essential trace element for humans due to the wide range of physiological functions for which it is needed. It is one of the many dietary minerals essential for free radical defense, especially for DNA oxidative damage prevention [115]. Just as with zinc, copper serves as a cofactor for some enzymes involved in host antioxidant defense, such as superoxide dismutase (SOD), which is the main direct defense against oxidative stress. This trace element is also required for collagen and elastin synthesis, both key factors for tissue integrity maintenance [116,117].

Cu can boost the host’s immune system capacity to counteract pathogens, exhibiting antiviral, antibacterial, and even antifungal effects. It has demonstrated a role in the development and maintenance of both innate and adaptive immune responses [118]. Under inflammatory conditions, patients show higher levels of serum copper and caeruloplasmin (the protein in charge of the carrying circulating Cu); hence, the increase in this element’s concentration may be associated with a physiological reaction performed so as to fight inflammation [119]. Regarding innate immunity, Cu plays a key role in the optimal functioning of several components of the immune system, supporting the activity of monocytes, macrophages, neutrophils, and NK cells. During infections, macrophages appear to employ high concentrations of copper as a defense mechanism since this would result in being toxic for invading pathogens [118]. It has been demonstrated that Cu can induce autophagy and apoptosis, collaborating in a cell’s antiviral defense [116,117]. As for adaptive immunity, copper is involved in T cell proliferation and response [65], as it has been associated with the promotion of IL-2 production. Some studies have shown that humans on a low copper intake had reduced IL-2 levels, together with decreased lymphocyte proliferation, which could be reversed through copper administration [82]. In addition, this trace element is also related to B cells’ functioning and antibody generation [120].

Copper deficiency impairs a number of immune functions, so it has been widely associated with altered and weakened responses, and consequently with an increased frequency and virulence of bacterial and viral infections [121]. The symptoms of this deficiency in humans include abnormalities in white blood cells, affecting both innate and adaptive lines, together with alterations in bone and connective tissues. These adverse effects seem to be more pronounced in two groups of the population: infants and the elderly. Infants with genetic disorders resulting in Cu deficiency are more prone to suffering from frequent and severe infections [122]. This deficiency can also over-activate neutrophils, causing their accumulation in the liver, and eventually contributing to a worse inflammation state [116]. Moreover, even a suboptimal intake (without reaching a critical level of deficiency) has been shown to be likely related to decreased T lymphocyte proliferation and affectation of phagocytosis in macrophages and neutrophils [98,122]. This relationship between Cu and immunity is supported by a meta-analysis of studies performed in Chinese children, which reported low hair copper content in those who experienced recurrent respiratory tract infections [16]. On the other hand, an extreme situation related to copper disturbance is Menkes syndrome, a rare congenital disease whose patients are characterized by a complete absence of caeruloplasmin. Children with this condition show immune impairments, and therefore, an increased susceptibility to infections, including pneumonia [82]. It must also be highlighted that, while severe copper deficiency has demonstrated an adverse influence in immune responses, the effects of insufficiency in humans are yet to be fully elucidated.

As stated before, supplementation has been proven to reverse the consequences of low Cu status, mainly through a restoration of IL-2 secretion and activity. This would also normalize the balance between Th1 and Th2 cells, which would have been affected by copper deficiency [116]. However, if needed, interventions should be carried out cautiously, as an excessive intake of Cu and high levels of serum concentrations have been associated with adverse effects not only on the immune system but also on respiratory health [123]. Copper toxicity is related to serious adverse responses, including gastrointestinal and urinary hemorrhage, and severe multi-organ injury, so attention should be paid to avoid such an outcome [117].

On its own, copper has demonstrated a potent ability to neutralize several types of enveloped/non-enveloped, single/double-strained RNA and DNA viruses. ROS seem to be involved in this mechanism of Cu-induced viral killing, with an essential role played by Cu+ and hydrogen peroxide [124]. On the other hand, the exposure of coronavirus 229E to Cu has been shown to irreversibly destroy its genome (by binding between and within its strands), deteriorate mRNA and capsid proteins involved in the viral life cycle, and affect its morphology. This last feature translates into changes that include envelope disintegration and spikes dispersal, which leads to the inhibition of viral entry [38,116]. It has been reported that enveloped viruses e more sensitive to Cu^2+^ than non-enveloped viruses, and the same has been observed for RNA viruses in comparison to those with DNA as a genome. Furthermore, Cu^2+^ can inhibit RNA polymerase activity by more than 60% [119]. Regarding SARS-CoV-2, it is quite sensitive to copper, which has been reported by an in vitro study in which Cu^2+^ blocked papain-like protease (PL-pro), a crucial protein for virus replication [125]. This is the reason why the combinations of Cu and other drugs, such as N-acetylcysteine or remdesivir, have been identified as candidates for COVID-19 pharmacological treatment [119]. On a different note, intravascular thrombosis is one of the most frequent causes of death in COVID-19 patients. In relation to this, some studies carried out in rats have shown that hemostatic function is impaired during copper deficiency, even though the mechanism of the formation of thrombotic lesions remains unclear [117].

**Figure 4:**
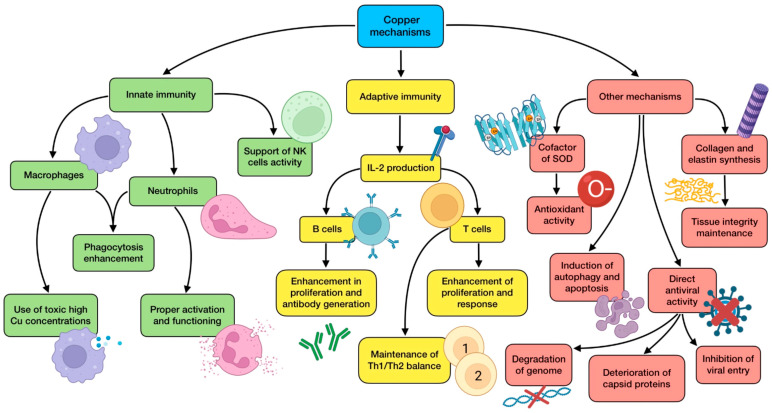
Summary of copper’s main mechanisms of action (references detailed in brackets). Through them, this trace element exerts several effects related to innate immunity [116,117,118,119,122], adaptive immunity [65,82,116,118,120,122], as well as other mechanisms associated with antioxidant defense [116,117], autophagy induction [116,117], direct antiviral activity [38,116,119,124,125], and tissue integrity maintenance [116,117]. Color description: green (innate immunity), yellow (adaptive immunity), and red (other mechanisms).

Owing to its ubiquitous distribution and the low amount needed, copper deficiency is rather uncommon. Adults usually meet its daily dietary recommendations as a result of a proper adherence to healthy eating guidelines. However, this does not always happen, especially in the elderly population due to malnutrition or malabsorption, and also when it comes to seriously ill people in ICUs receiving parenteral nutrition [39]. In fact, Lee et al. [40] assessed the clinical significance of trace elements’ serum levels in 167 critically ill patients and found that low Cu concentrations were observed at ICU admission, and higher levels during an ICU stay were associated with a significantly lower mortality rate. Copper deficiency is not always a result of a lack of copper, but can also be the consequence of an imbalance between this and other trace elements in supplemented diets, for instance, due to high zinc intakes. As Zn and Cu are competitively absorbed in the jejunum via metallothionein, excessive zinc doses (>150 mg/day) can lead to copper deficiencies in healthy people. This should be taken into consideration when a COVID-19 patient is taking a regular Zn supplementation to boost their immune system against the virus [116]. Currently, there is not enough knowledge to recommend a dietary intake of this nutrient specifically against COVID-19, but a Cu intake of 7.8 mg/day has proved to be enough to maintain optimal plasma levels so as to improve innate and adaptive immunity and reduce oxidative stress, which would be preventively and/or therapeutically useful against SARS-CoV-2 [126]. In conclusion, a controlled and proper supplementation to correct its deficit might be advisable due to its benefits for COVID-19 patients, especially critically ill ones [116]. In addition, alterations found in serum biomarkers of Cu and Se status in COVID-19 seem not to be compatible with a simple acute phase response, contributing their levels to a good prediction of disease survival [41]. Consequently, it is important to increase research in this field to achieve a greater understanding that allows determination of what intake of dietary or supplemented Cu might be beneficial against the disease. Figure 4 summarizes the mechanisms of action performed by copper.

### 3.5. The Role of Magnesium (Mg)

Magnesium is one of the most abundant cations in the organism. It acts as a cofactor or activator for more than 600 enzymes involved in several biochemical reactions and pathways related to a cluster of physiological functions, including energy metabolism, protein and nucleic acid synthesis, and potassium and calcium transport. As for serum magnesium, about 32% is bound to albumin, and about 55% is present as free ionized cation iMg(2+), which is the bioactive form of this element. Whole blood iMg(2+) has been proposed as a more sensitive measure of magnesium status in comparison with total serum or urinary levels [127]. Clinical magnesium deficiency owing to low dietary intake is not relatively frequent in otherwise healthy subjects since kidneys are able to tightly regulate its urinary excretion. However, latent subclinical deficiency is a more common situation in these populations. Hypomagnesemia usually goes unrecognized in clinical medicine, as magnesium concentrations are rarely monitored in routine procedures, and can be present despite normal serum Mg levels. Nevertheless, it is a frequent feature in hospitalized patients, especially among critically ill ones. Even though a severe state of deficiency with clinical symptoms is rare, whenever it takes place, it can be a serious and even potentially fatal complication if not addressed [128,129].

With regard to the role of this mineral in immune responses, there are several associations with both innate and adaptive immunity that deserve to be highlighted. Low Mg intake has shown an inverse relationship with a number of markers of systemic inflammation and endothelial dysfunction, including IL-6, TNFα, CRP, soluble intercellular adhesion molecule 1 (sICAM-1), soluble vascular cell adhesion molecule 1 (sVCAM-1), and other acute phase proteins [128]. Therefore, Mg deficiency has been related to a chronic low-grade state of inflammation, which would exacerbate eventual virus-induced inflammation, aggravating the uncontrolled release of pro-inflammatory cytokines. One of the proposed mechanisms of action of Mg that may explain these findings is related to its “calcium-channel blocking” effect. Magnesium can inhibit calcium influx in immunocompetent cells, leading to a reduction in NF-κB activation, which results in lower cytokine production and release (especially IL-6), and therefore, less systemic inflammation [130]. As for adaptive immunity, magnesium-deficient conditions have been shown to reduce in vitro and in vivo proliferation and activation of both CD4+ and CD8+ T cells. Interestingly, CD8+, and to some extent CD4+ lymphocytes, are significantly decreased in the lungs of mice affected with this mineral deficiency, in which inhalation of the influenza A virus resulted in exacerbated morbidity [42]. This suggests that a low Mg status could support the lymphocyte impairment reported in other viral infections, such as COVID-19.

This situation could be reversed, as systematic reviews of randomized controlled trials have reported an inverse correlation between CRP levels and magnesium supplementation [127]. Co-supplementation with zinc has also shown a similar outcome regarding CRP levels, together with a down-regulation of genes that control TNFα and IL-1 expression, and an improvement in total plasma antioxidant capacity [43]. In addition, a short-term administration of magnesium sulfate has been shown to effectively attenuate the production of TNFα and IL-6 by monocytes under TLR-stimulated conditions, also being able to inhibit other inflammatory components such as macrophage inflammatory protein-2, prostaglandin E2, and cyclooxygenase-2 in lung tissue. On the other hand, intravenous infusion of Mg has been proven to have a role in attenuating superoxide (O^2−^) production in neutrophil respiratory burst [129,131].

Magnesium is also related to vitamin D, which has been reported to reduce the risk of infection through several mechanisms of action [132]. This mineral is used as a cofactor in multiple steps of vitamin D metabolism, including 25(OH)-D and 1,25(OH)-D synthesis, binding of vitamin D to vitamin-D-binding protein (VDBP), and vitamin D receptor (VDR) activation. The amount of VDRs in target cells is reduced during Mg deficiency, leading to an attenuation in the vitamin D effects mediated by them. In addition, 1,25(OH)D serum levels frequently remain low in subjects with deficiency of this mineral regardless of vitamin D intake, highlighting a noticeable association [133]. As for COVID-19, hypomagnesemia is present in most of the patients with moderate to severe cases. Some characteristics of the disease recall the signs and symptoms described in Mg deficiency, so it is reasonable to hypothesize that magnesium deficiency may contribute to the appearance, onset, progression, and seriousness of SARS-CoV-2 infection [127]. As has previously been stated, subjects with comorbidities, including CVD, hypertension, obesity, and diabetes, are more prone to developing a severe course of the disease, and all these comorbidities have been related to a state of hypomagnesemia, maybe exacerbated by some of the drugs used to treat them [134].

Magnesium is a relevant nutrient for cardiovascular health. It inhibits smooth muscle contraction, being able to reduce blood pressure (including systolic, diastolic, and mean values) probably by blocking the release of calcium from the sarcoplasmic reticulum, promoting its outflow, and increasing nitric oxide (NO) levels [135]. This mineral is also relevant for maintaining endothelial function and vascular integrity. The pro-inflammatory phenotype induced by Mg deficiency leads to increased thrombogenicity owing to the augmented release of cytokines and chemokines. The endothelium responds to the inflammatory stimuli by releasing von Willebrand factor, which favors the bond of platelets to arterial walls. Concurrently, Mg deficiency also induces platelet aggregation and the release of thromboxane and thromboglobulin from them. Furthermore, low magnesium status also affects fibrinolytic activity through an up-regulation of type 1 plasminogen activator inhibitor [131]. Consequently, these findings point to the fact that Mg deficiency might create a favorable microenvironment for thromboembolism to be promoted. Other findings point out that magnesium supplementation can also reduce the risk of atrial fibrillation and improve subclinical atherosclerosis [135]. Therefore, the administration of this element to correct a deficient status would be beneficial to ameliorate COVID-19 comorbidities, as well as the cardiovascular complications of the disease.

Magnesium is also involved in maintaining proper lung function. It inhibits bronchial smooth muscle contraction, thus reducing the risk of airway hyperreactivity. In addition, it may help prevent lung fibrosis by preventing the deposition of collagen in lung tissue since it can decrease the release of transforming growth factor-β1 (TGF-β1) [44]. In a model of acute lung injury, the administration of magnesium sulfate ameliorated lung histopathology, reducing inflammatory cell infiltration, alveolar edema, and alveolar exudation, and also balancing oxidative stress [129]. These are relevant features in respiratory tract infections, including COVID-19, suggesting Mg as an adjuvant treatment that would be beneficial for controlling and ameliorating symptoms in lung pathologies. A great body of scientific evidence on these pathologies supports this assumption. Abnormal Mg status on admission was found to correlate with an increased mortality rate in adults with community-acquired pneumonia [45]. A transversal study on COVID-19 reported that the risk of magnesium deficiency was significantly and negatively related to infection severity, oxygen therapy, and ICU stay. This research also revealed a high prevalence of hypomagnesemia in COVID-19 hospitalized patients [46]. The administration of intravenous magnesium sulfate to hospitalized children with acute severe asthma significantly improved their respiratory function and reduced the number of subjects requiring mechanical ventilation [136]. Cumulative evidence from the meta-analysis of clinical trials keeps on highlighting the benefits of Mg supplementation on lung illnesses [129]. For instance, results from a cohort study carried out with old COVID-19 patients showed that a combined treatment consisting of magnesium (150 mg daily), vitamin D, and vitamin B12 significantly reduced the need for oxygen and/or ICU support [47,129]. On the other hand, 12-week personalized Mg treatment among a <65 years old population led to methylation changes in the transmembrane serine protease 2 (TMPRSS2) gene, which codifies a host-expressed protein required for SARS-CoV-2 entry. This finding confirms another potential role of magnesium intervention in COVID-19 prevention [48].

**Figure 5:**
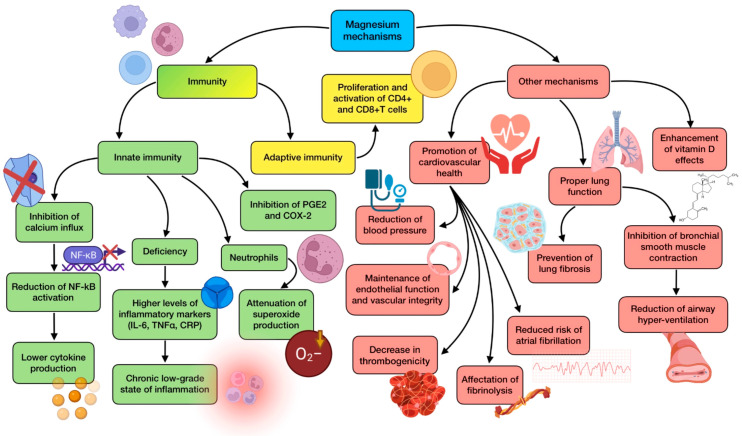
Summary of magnesium’s main mechanisms of action (references detailed in brackets). Through them, this mineral exerts several effects related to innate immunity [128,129,130,131], adaptive immunity [42], as well as other mechanisms associated with cardiovascular health [131,134,135], lung function [44,45,129], and vitamin-D-related mechanisms [132,133]. Color description: green (innate immunity), yellow (adaptive immunity), and red (other mechanisms).

Taking all these findings into consideration, increasing the dietary intake of magnesium through both food and modest supplementation could be a safe, inexpensive, and available intervention during the early stages of the disease, exerting a potential benefit for patients with very limited risk because of its large enough safe window. Mg infusions would present a greater risk but would also allow a quick repletion when it comes to emergency situations, such as the beginning of a cytokine storm. ICUs may consider this magnesium administration in COVID-19 patients with suspected hypomagnesemia, providing a dose of 1 g of intravenous Mg every 6 h, and taking the precaution of not exceeding renal excretory capacity. Healthy individuals could consider a daily supplementation with 350–400 mg of magnesium in order to prevent the infection, and twice a day if mild symptoms have just appeared, especially when dietary intake is lower than recommended [127,129]. Mg supplementation would certainly protect tissues and organs from damage by performing several mechanisms related to immune regulation, anti-inflammation, and antioxidation. Nevertheless, more basic, translational, and clinical research is needed to provide more evidence that will help to unravel the potential benefits of a proper Mg status during COVID-19. A summary of magnesium’s main mechanisms of action is detailed in Figure 5.

### 3.6. The Role of Iron (Fe)

Iron has a role in many biological functions, such as systemic oxygen transfer through hemoglobin. This essential trace element acts as an electron donor or acceptor in several enzymatic systems, including CYP450 and electron transport chain. It is a necessary component for cell growth, differentiation, and functioning, as it is essential for DNA synthesis through ribonucleotide reductase. However, alteration in iron homeostasis is also associated with inflammation and oxidative stress at a systemic level [137]. The structure of iron makes it play an essential role as a mediator of oxidative stress since it acts as a redox catalyst. One of these actions is related to its ability to generate hydroxyl radicals, highly toxic for pathogens, which translates into a powerful antimicrobial effect. The trace element is required for several immune processes, being a crucial component of different enzymes, which are involved in essential immune cells’ functions [49]. For instance, Fe regulates cytokine production, enhances neutrophil activity against microorganisms, and allows T cell proliferation and maturation [138].

The prevalence of iron deficiency worldwide is rather high, with its relation to infectious diseases being well known and recognized. Deficiency, especially for prolonged periods of time, has been associated with wide-ranging effects, including reduced antibody generation by B lymphocytes [65], compromised cytokine production, decreased activity of NK cells [49], thymus atrophy, disturbance in the output of naive T cells, reduced T lymphocytes’ proliferation (about 50–60% compared to iron replete subjects), difficulties in Th1 cells’ cytokine production, and respiratory burst impairment [82]. These findings suggest that iron deficiency could clearly raise the host’s susceptibility to infections, including the respiratory ones, in which the role of iron has been critically studied and reviewed, for both bacterial and viral diseases [139]. Alterations in iron uptake and metabolism are involved in the virulence of acute and chronic respiratory infections. In addition, low serum levels of iron have been related to dampened lung function [98].

Despite all of this, iron administration during infections could turn detrimental owing to different reasons. Firstly, even though iron is necessary for an optimal immune response, its overload leads to immune function impairment and damaging inflammation [140]. Secondly, microbes need iron for their growth and development, so providing it may favor their establishment and the harmful consequences derived by them. This is the reason why some host defense mechanisms are focused on withholding iron from microorganisms [141]. Thirdly, an excess of iron content in tissues has been associated with harmful cellular toxicity, especially concerning the lungs, with pulmonary inflammation and impaired function [98]. Therefore, it is important for serum iron concentrations to be well regulated and controlled during infections, monitoring its intake, and following the established recommendations. In this sense, studies are contradictory when it comes to elucidating the relevance of supplementation. A case–control study carried out in 485 hospitalized children (2–5 years old) who were provided with iron supplementation for 3 months, concluded that the recurrence of respiratory tract infections was decreased in a significant way [50]. However, a South African study performed with iron-deficient schoolchildren concluded that supplementation increased the risk of respiratory infections [142]. Within the context of COVID-19, in iron-deficient patients who were stabilized after acute heart failure, treatment with ferric carboxymaltose decreased the hazard of heart failure hospitalizations, without an apparent effect on cardiovascular death risk [51]. This trace element has also been tested for immune boost during COVID-19 vaccination through intravenous supplementation. However, even though this intervention efficiently restored iron status in the subjects of the study, the humoral and cellular immune response against the virus did not improve after three doses of vaccination [52].

Iron metabolism is clearly disrupted by SARS-CoV-2, especially in those subjects who are more affected by the disease. Anemia and hyperferritinemia have been reported as typical manifestations in hospitalized patients [53]. A retrospective study carried out with subjects from 50 Chinese hospitals showed that 90% of them exhibited abnormally low serum Fe levels, also showing a correlation between this situation and disease severity [54]. Despite this fact, blind supplementation can be a double-edged sword, as it may aggravate hyperferritinemia and its consequences. Ferritin allows iron storage in its ferric state (Fe^3+^). A single molecule of ferritin can carry more than 4000 iron atoms in its core [137]. When it comes to COVID-19, serum ferritin levels in non-survivors have been demonstrated to be twice as high as those in survivors, which suggests that hyperferritinemia is a significant predictor of increased mortality risk [143]. However, even though a strong association has been observed, it remains unclear whether hyperferritinemia is merely a systemic marker of COVID-19 progression or an important modulator of its pathogenesis. Broadly speaking, systemic inflammation is related to augmented ferritin serum levels, as some cytokines such as IL-6, whose secretion has been demonstrated to be increased during the COVID-19 hyperinflammatory state, are able to stimulate not only ferritin but also hepcidin synthesis [144]. Hepcidin is a key molecule in iron homeostasis regulation, and it seems to have a relevant role during infections and inflammation. It exerts several functions: down-regulating iron absorption to limit the available pool of this element available for pathogens; sequestering iron within different cells, such as enterocytes and macrophages; and preventing iron efflux from them. This consequently leads to increased intracellular ferritin so as to store it [144].

The excess of intracellular iron makes contact with oxygen molecules, which eventually generates ROS through Fenton and Haber–Weiss reactions, together with RNS and even RSS. As a consequence, ferroptosis takes place, which is a process of programmed cell death mediated by iron-dependent peroxidation mechanisms, leading to further tissue damage [145]. The oxidative damage would affect cellular components of several organs (lungs, liver, kidney, and heart). Ferroptosis has also been associated with neurological disturbances, including cognitive impairment [145], anosmia, and ageusia [55], which are frequent manifestation of SARS-CoV-2 infection. This iron overload might also affect lung and gut microbiota diversity, and the blood coagulation process [137,146], which needs to be taken into consideration as coagulability has been shown to be a major concern in COVID-19 pathogenesis. Excessive ferritin leakage from damaged tissues consequently releases free iron. When this iron is in its oxidized state (Fe^3+^), it can accelerate serum coagulation through an interaction with different proteins of the coagulation cascade. As a result, the risk of a cardiovascular event is significantly increased [137].

Histological analyses of COVID-19 patients have reported diffuse endothelial inflammation with thrombosis, edema, and ischemia. One of the proposed mechanisms possibly involved in these clinical findings is the dysregulation of iron homeostasis. These disturbances, attached to higher iron levels, may support the progression of viral infections, characterized by ADRS and pulmonary fibrosis. The excessive oxidative and nitrosamine stress mentioned above has been proposed as contributing to the pathogenesis of ARDS. Patients with pulmonary fibrosis are characterized by elevated iron accumulation in the lungs, where fibroblasts exhibit a high proliferation and cytokine response. This is why assessing patients’ ferritin levels might help predict the course of ARDS, thus enhancing their treatment [56]. Notably, iron homeostasis can also be altered by SARS-CoV-2 in a different way. An in silico analysis carried out on viral protein sequences has shown that some of them are able to disturb human heme metabolism since they may generate a complex with porphyrin and affect the 1-β chain of hemoglobin, which would result in the dissociation of the iron atom [147].

Iron chelators, such as deferoxamine (DFO), deferasirox, and deferiprone, could be a treatment alternative in these cases, having already been approved for the treatment of iron overload. Moreover, owing to its immunomodulatory properties, DFO is able to inhibit the development of invading microbes, including viruses, although its benefits concerning COVID-19 are not elucidated yet. These properties are mainly associated with an increase in B cell levels and neutralizing antibody titers [148]. In addition, reduced iron availability generated because of chelators’ activity would also play an important role in the reduction of SARS-CoV-2 replication, as has been reported before in several other RNA viruses. Administration of DFO has also been shown to improve the response to IFN-α treatments [147]. On the other hand, it has also been suggested that iron chelators may come in handy as a way to prevent the appearance of pulmonary fibrosis and lung function decline following SARS-CoV-2 infection, which has been related to iron dysregulation and increased levels [56]. As for endothelial inflammation, DFO has been reported to suppress it during influenza A infections by inhibiting IL-6 production through a decrease in NF-kB activity [147], so it could also play a role in the COVID-19 cytokine storm.

**Figure 6:**
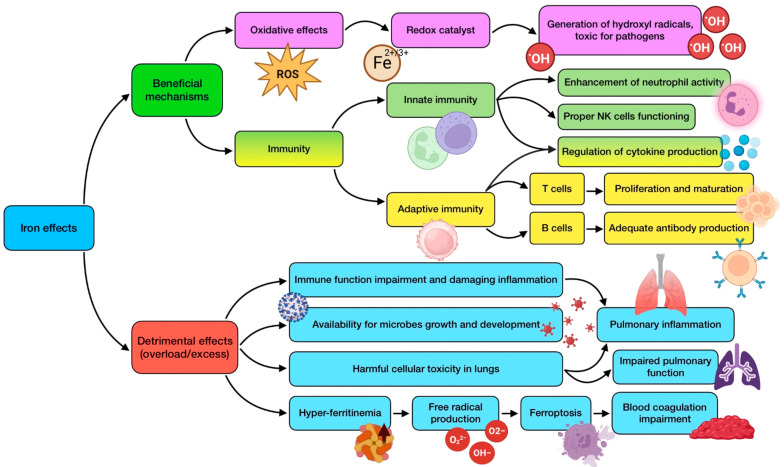
Summary of iron’s beneficial mechanisms of action and detrimental effects derived from its overload or excess (references detailed in brackets). Through these mechanisms, this trace element exerts several effects related to antioxidant defense [49], innate immunity [49,138], and adaptive immunity [65,82,138]. Its potentially negative effects are associated with immune function impairment [141], availability for microbes [141], cellular toxicity in lungs [98], and hyperferritinemia/ferroptosis [55,137,144,145,146]. Color description: purple (antioxidant defense), green (innate immunity), yellow (adaptive immunity), and blue (detrimental effects).

In conclusion, the relevance of iron in infectious diseases makes it crucial to investigate the different iron-related parameters in COVID-19 patients, including serum iron levels, hepcidin, transferrin saturation, and non-transferrin bound iron. A situation of increased transferrin saturation may reflect a state of iron overload, which should be considered in order to decide the best intervention. Therefore, considering the strategy of targeting the intracellular iron overload would be advisable in future controlled clinical trials. As for possible adjunctive treatments, iron chelators, ferroptosis inhibitors, and hepcidin modulators should be further investigated [137]. Figure 6 summarizes the most relevant effects of iron, highlighting its mechanisms of action against infections as well as its potential negative effects due to overload or excess of this trace element.

### 3.7. The Role of Other Minerals

Sodium (Na): this macro-mineral seems to play a relevant role in the expression of ACE2 in SARS-CoV-2 [149]. It has been found that sodium levels are significantly reduced in COVID-19 patients, and this hyponatremia decreases with the severity of the disease [57].

Potassium (K): a situation of hypokalemia can increase the risk of ARDS and acute cardiac injury, which are two of the most frequent COVID-19 complications. The reduction of ACE2 expression, following the binding of SARS-CoV-2 to its receptor, translates to an increase in angiotensin II release, subsequently leading to hypokalemia and related complications [149]. Severe COVID-19 patients have shown significantly lower potassium concentrations than non-severe ones [57].

Calcium (Ca): this mineral collaborates in the elimination of invading viruses from cells, having demonstrated an ability to protect subjects against the common cold. Calcium concentrations have been reported to be lower in critical COVID-19 patients, with these levels being inversely proportional to the severity of the illness [58]. Just as with low sodium or potassium levels, hypocalcemia may also act as a marker of the seriousness of the infection.

Manganese (Mn): this trace element possesses antioxidant activity since it acts as a cofactor of the human Mn superoxide dismutase (Mn-SOD), which is one of the main mitochondrial antioxidants [149]. Manganese also exerts some immunomodulatory and antiviral effects, including the activation of IFN signaling, the amelioration of CD8+ T cell memory, or the induction of M1 macrophage polarization. These activities seem to be improved by using a Mn nanodepot (nano-Mn) [59]. Evidence also points out that Mn deficiency might be associated with impaired antibody production [150].

Iodine (I): some iodine-based products such as povidone-iodine (PVP-I) have been suggested as possible chemical agents against SARS-CoV-2, being as efficient as 70% ethanol. Consequently, it may be applied as a disinfectant for hand washing, spraying the throat, gargling, or other external uses. However, iodine also has a role as an antiviral in in vivo systems, including respiratory mucosa, airways, and saliva. Innate antiviral immunity and mucosal oxidative defense are augmented by this trace element, which has also been shown to be relevant as a way to reduce de severity of the respiratory syncytial virus [149].

Cobalt (Co): it is essential for blood cells’ formation as it is a crucial component of vitamin B12. Some studies have revealed that a complex of cobalt (III) can hydrolyze phosphodiester bonds in viral DNA and RNA [149]. In addition, its ability to block RNA translation due to its high affinity towards it, provides this trace element with therapeutic effects against a number of viruses, such as the herpes simplex virus and Epstein–Barr virus [151].

Sulfur (S): it is necessary to produce essential amino acids cysteine and methionine, which are relevant in bio-catalytic processes, immune activities, and blood clotting [149]. It has been reported that sulfate-based compounds exert therapeutic efficacy during respiratory infections. For instance, sodium thiosulfate can ameliorate the pneumonia course and lung injuries in both children and adults [152], possibly having a protective role in COVID-19.

Lithium (Li): this element exhibits antiviral effects against some coronaviruses, reducing its replication, cellular entry, and the transcription and translation of viral proteins [125]. Lithium also exerts some anti-inflammatory activities, including the reduction of IL-1β and TNFα, the augmentation of IL-2 and IL-10, and the inhibition of COX-2 expression [153].

Nickel (Ni): it is required to some extent for the modulation of the immune system, being able to enhance B and T cells’ activity in the spleen, while reducing NK cells’ function [153].

### 3.8. Limitations of the Study

One of the main limitations of this review has been the heterogenicity of the studies included in it. The incorporation of several types of studies answers to a lack of solid scientific knowledge derived from randomized clinical trials, which, nevertheless, have been preferential in the search process. On the other hand, due to the difficulty of finding studies specifically involving these elements and COVID-19, studies about other similar respiratory diseases have also been taken into consideration. This has consequently complicated the conclusion-making process, as well as limited the potential of the conclusions drawn strictly regarding SARS-CoV-2 infection. Another relevant limitation has been the controversy between the featured results in different studies about the same topic, which has made it difficult to draw accurate conclusions from them. Finally, minerals different from zinc, selenium, copper, magnesium, and iron have been studied in a very limited way when it comes to COVID-19 and similar diseases, so they have been gathered in a common section of the review that outlines the most relevant findings associated with them.

## 4. Conclusions

There is scientific evidence related to COVID-19 and similar respiratory diseases pointing out that minerals may play a relevant role in preventing this illness and ameliorating its course and progression. Immuno-mediated effects are wide and common among the minerals reviewed, especially zinc, selenium, copper, and magnesium. They regulate the activity of both innate and adaptive immunity, enhancing the functioning of a wide range of immune cells, and modulating cytokine expression and release, something extraordinarily relevant during SARS-CoV-2 infection. The antioxidant benefits derived from these minerals are also noteworthy, being associated with zinc and copper through superoxide dismutase, and with selenium through a variety of selenoproteins. Tissue integrity maintenance, barrier enhancement, and direct antiviral activity are some other roles that have been related to some trace elements such as zinc and copper. A tight relationship with the renin–angiotensin system through ACE2 expression has been associated with zinc, and cardiovascular and pulmonary benefits have been observed due to magnesium. Supplementation of these minerals might be considered, but interactions between some of them such as zinc and copper must be taken into consideration before any intervention. Less clear is the role of iron due to the need that pathogens have for this element and the consequences derived from its excess; so the use of iron chelators may be a treatment alternative in some cases of disease. Other trace elements such as manganese, iodine, cobalt, and even electrolyte status have been less addressed by the scientific community, but they exert mechanisms potentially relevant in COVID-19 that deserve further investigation. Therefore, minerals may represent an important strategy for the prophylaxis and management of the disease. Nevertheless, there is a lack of conclusive and well-designed clinical trials that demonstrate the benefits of supplementation during COVID-19, so further research is needed. These micronutrients could represent a cheap and easily applicable adjuvant therapy, not only to prevent and correct nutritional deficiencies, which is always advisable, but also to boost the physiological mechanisms that help the organism fight the infection.

## Figures and Tables

**Table 1:** Articles included and reviewed.

Reference	Mineral	Study Design	Major Findings
Wessels I et al., 2020 [10]	Zinc	Animal model	Zinc supplementation improves lung injury through a reduction in neutrophil recruitment and activity.
Jothimani D et al., 2020 [11]	Zinc	Observational	A significant number of COVID-19 patients are zinc deficient, they develop more complications, and the deficiency is associated with a prolonged hospital stay and higher mortality rate.
te Velthuis AJ et al., 2010 [12]	Zinc	In vitro	Zn(2+) inhibits coronavirus RNA polymerase activity and its ionophores block their replication in cell culture.
Roscioli E et al., 2017 [13]	Zinc	In vitro	Zinc deficiency as a codeterminant for airway epithelial barrier dysfunction through an increase in its permeability.
Hemilä H, 2017 [14]	Zinc	Meta-analysis	Zinc lozenges are useful for common cold treatment, significantly reducing its duration.
Science M et al., 2012 [15]	Zinc	Meta-analysis	Oral zinc formulations shorten the duration of symptoms of the common cold.
Mao S et al., 2014 [16]	Zinc, Copper, Iron	Meta-analysis	Zn, Cu, and Fe deficiency may be contributing factors for the susceptibility of recurrent respiratory tract infections in children.
Anuk AT et al., 2021 [17]	Zinc, Copper, Magnesium	Observational	Pregnant women with COVID-19 show lower levels of serum zinc and Zn/Cu ratio, as well as higher levels of serum copper and magnesium. Serum zinc and Zn/Cu ratio concentrations had a negative relationship with acute phase markers such as IL-6 and CRP.
Heller RA et al., 2021 [18]	Zinc, Selenium	Observational	Zn and SELENOP status within the reference ranges is related to a higher survival odd in SARS-CoV-2 infection, and correcting a proven deficit in these trace elements by a personalized supplementation supports recovery.
Ben Abdallah S et al., 2023 [19]	Zinc	Clinical trial	Oral zinc can reduce 30-day death, ICU admission rate, and hospital stay, also shortening symptom duration in COVID-19 patients.
Reino-Gelardo S et al., 2023 [20]	Zinc, Selenium	Clinical trial	The administration of a food supplement in severe COVID-19 patients has protective effect, allowing early recovery from digestive symptoms and shorter hospital stay.
Natarajan S et al., 2021 [21]	Zinc	Clinical trial	In patients without clinical symptoms and comorbidities, viral load was significantly reduced on the seventh day after zinc and vitamin C supplementation, without progression to a symptomatic state.
Finzi E et al., 2020 [22]	Zinc	Case reports	All COVID-19 patients experienced relevant improvement after one day of high-dose zinc therapy.
Patel O et al., 2021 [23]	Zinc	Clinical trial	The use of high-dose intravenous Zn in hospitalized COVID-19 patients appears safe and feasible. No outcomes nor conclusions about recovery or ventilation need were drawn due to a lack of enrolment.
Abdelmaksoud AA et al., 2021 [24]	Zinc	Clinical trial	Zinc therapy might have a significant role in reducing the duration of smell and taste recovery in COVID-19 patients, without affecting the total recovery duration from the disease.
Stambouli N et al., 2022 [25]	Zinc	Clinical trial	A significant decrease in SARS-CoV-2 infection among healthcare professionals is reported when doxycycline is administered with zinc instead of just the drug.
Rodriguez JAM et al., 2021 [26]	Zinc, Selenium	Clinical trial	Mean levels of CD4+T, CD3+T, and CD8+T cells increase after the second dose of the COVID-19 vaccine due to the administration of a combination of β-glucans with Se- and Zn-enriched Saccharomyces cerevisiae. An augmentation in both IgG and IgM is also reported.
Thomas S et al., 2021 [27]	Zinc	Clinical trial	Ambulatory COVID-19 patients treated with high-dose zinc gluconate, ascorbic acid, or a combination of both, do not show a significant decrease in the duration of symptoms.
Abd-Elsalam S et al., 2021 [28]	Zinc	Clinical trial	Zinc supplementation in COVID-19 hospitalized patients did not improve the clinical efficacy of hydroxychloroquine.
Maywald M et al., 2017 [29]	Zinc	In vitro	Zinc can ameliorate the allogeneic immune reaction by enhancing antigen-specific iTreg, so it can be considered as a possible tool for inducing tolerance in adverse immune reactions.
Wang Y et al., 2021 [30]	Selenium	In vitro	SARS-CoV-2 suppresses mRNA expression of several selenoproteins involved in ferroptosis, endoplasmic reticulum stress, and DNA synthesis.
Lee YH et al., 2016 [31]	Selenium	Observational	Low selenium levels in patients suffering from respiratory diseases have a major correlation with poor nutritional status and prognosis on admission.
Tseng CK et al., 2013 [32]	Selenium	Observational	Serum selenium is inversely associated with inflammatory cytokine IL-6 in the elderly.
Gordon DE et al., 2020 [33]	Selenium	In vitro	SARS-CoV-2 viral proteases such as M-pro are able to induce the degradation of host selenoproteins.
Jin Z et al., 2020 [34]	Selenium	In silico	An organoselenium compound called “ebselen” shows a strong inhibitory activity against SARS-CoV-2 protease M-pro. It also performs as an important suppressor of PL-pro.
Mahmoodpoor A et al., 2019 [35]	Selenium	Clinical trial	Selenium administration restores lung antioxidant capacity, moderates inflammatory responses, and improves respiratory mechanics in critically ill patients with ARDS, not showing a relevant effect on overall survival, duration of mechanical ventilation, and ICU stay.
Zhang J et al., 2020 [36]	Selenium	Observational	There is a positive correlation between selenium concentrations and COVID-19 recovery rate.
Moghaddam A et al., 2020 [37]	Selenium	Observational	Selenium status was significantly higher in surviving COVID-19 patients compared with non-survivors, showing a relationship between selenium levels and mortality risk in this disease.
Warnes SL et al., 2015 [38]	Copper, Zinc	In vitro	Human coronavirus 229E is rapidly inactivated on a range of copper alloys, and Cu/Zn surfaces are very effective at lower copper concentrations.
Wazir SM et al., 2017 [39]	Copper	Case reports	Copper deficiency is associated with anemia, leucopenia, and myeloneuropathy.
Lee YH et al., 2019 [40]	Copper, Zinc	Observational	At the time of ICU admission, serum concentrations of copper and zinc are lower than the normal values in critically ill patients. Serum levels measured on day 14 of ICU stay are higher than those measured at the time of ICU admission for zinc and copper, being associated with a significantly lower mortality.
Hackler J et al., 2021 [41]	Copper, Selenium	Clinical trial	Modifications in serum biomarkers of Cu and Se status during SARS-CoV-2 infection are not compatible with a simple acute phase response, copper and SELENOP serum concentrations contribute to a good prediction of disease survival.
Kanellopoulou C et al., 2019 [42]	Magnesium	Animal model and in vitro	Mg^2+^ regulates the active site of certain kinases during T cell responses, so keeping high serum Mg^2+^ concentration is important in order to boost antiviral immunity.
Afshar Ebrahimi F et al., 2018 [43]	Magnesium, Zinc	Clinical trial	Magnesium and zinc co-supplementation has beneficial effects on serum hs-CRP, oxidative stress markers, and gene expression of IL-1 and TNF-α.
Yang Q et al., 2019 [44]	Magnesium	Animal model and in vitro	Magnesium isoglycyrrhizinate attenuates pulmonary fibrosis partly by inhibiting fibroblast differentiation.
Nasser R et al., 2018 [45]	Magnesium	Observational	Hypomagnesemia and hypermagnesemia on admission were related to an increased rate of 30-day mortality among patients hospitalized with community-acquired pneumonia.
Quilliot D et al., 2020 [46]	Magnesium	Observational	The risk of Mg deficiency is negatively related to SARS-CoV-2 infection severity, oxygen therapy, and ICU stay in critical care unit. There is a high prevalence of hypomagnesemia in COVID-19 hospitalized patients.
Tan CW et al., 2020 [47]	Magnesium	Clinical trial	A magnesium, vitamin D, and vitamin B12 combination in older COVID-19 patients is associated with a relevant reduction in clinical deterioration requiring oxygen support, ICU support, or both.
Fan L et al., 2021 [48]	Magnesium	Clinical trial	Twelve-week personalized Mg treatment in the elderly led to methylation changes in TMPRSS2 gene, confirming another potential role of magnesium intervention in COVID-19 prevention.
Agoro R et al., 2018 [49]	Iron	Animal model	Iron modulates the inflammatory response outcome through an influence in macrophage polarization.
Jayaweera JAAS et al., 2019 [50]	Iron	Observational	Iron deficiency makes children more prone to develop acute respiratory tract infections. Once iron deficiency is corrected, the rate of recurrent infections is reduced.
Ponikowski P et al., 2020 [51]	Iron	Clinical trial	In iron-deficient patients who were stabilized after acute heart failure, treatment with ferric carboxymaltose decreases the hazard of heart failure hospitalizations, without an apparent effect on cardiovascular death risk.
Vinke JSJ et al., 2023 [52]	Iron	Clinical trial	Intravenous iron supplementation efficiently restores its status, but humoral and cellular immune response against SARS-CoV-2 does not improve after three vaccinations.
Bolondi G et al., 2020 [53]	Iron	Observational	Lymphopenia is severe and constant in COVID-19 critically ill patients. Transferrin saturation is extremely reduced at ICU admission, with the same trend observed for serum iron. Hyperferritinemia is constant during ICU stay.
Zhao K et al., 2020 [54]	Iron	Observational	Serum iron deficiency is detected in the patients with SARS-CoV-2 infection. The severity and mortality of COVID-19 is tightly correlated with serum iron concentrations. Low iron level is an independent risk factor for death in these patients.
Dinc ME et al., 2016 [55]	Iron	Observational	Patients with iron deficiency have an altered olfactory function with a significantly lower threshold, discrimination, and identification capacity.
Ali MK et al., 2020 [56]	Iron	Animal model	Augmented accumulation of pulmonary iron has a key role in the pathogenesis of pulmonary fibrosis and lung function affectation.
Lippi G et al., 2020 [57]	Sodium, Potassium, Calcium	Observational	COVID-19 severity is related to lower serum levels of electrolytes such as sodium, potassium, and calcium.
Rodriguez-Morales AJ et al., 2020 [58]	Calcium	Meta-analysis	Calcium concentration has reported to be lower in critical COVID-19 patients, with these levels being inversely proportional to the severity of the disease.
Sun Y et al., 2021 [59]	Manganese	Animal model	Manganese nanodepot boosts host immune response against coronavirus, offering a simple, safe, and robust strategy against the disease.

**Table 2:** Clinical trials involving mineral supplementation and COVID-19.

Reference	Mineral	Dose	Population	Major Findings
Ben Abdallah S et al., 2023 [19]	Zinc	50 mg/day of elemental Zn for 15 days	COVID-19 ambulatory and hospitalized patients	Oral zinc can reduce 30-day death, ICU admission rate, and hospital stay, also shortening symptom duration.
Reino-Gelardo S et al., 2023 [20]	Zinc, Selenium	1.5 mg/day of Zn and 8.25 µg/day of Se combined with vitamin D, probiotics, and prebiotics	Severe COVID-19 and hospitalized patients	The administration of a food supplement has protective effect, allowing early recovery from digestive symptoms and shorter hospital stay.
Natarajan S et al., 2021 [21]	Zinc	100 mg/day of Zn combined with ascorbic acid for 7 days	Positive COVID-19 patients without clinical symptoms	Viral load declines significantly after zinc and vitamin C supplementation, without progression to a symptomatic state.
Patel O et al., 2021 [23]	Zinc	0.24 mg/kg/day of elemental Zn 7 days maximum	COVID-19 hospitalized patients	Treatment with high-dose intravenous zinc appears safe and feasible, only being associated with minimal peripheral infusion site irritation.
Abdelmaksoud AA et al., 2021 [24]	Zinc	440 mg/day of Zn sulfate (50 mg elemental Zn)	COVID-19 ambulatory and hospitalized patients	Zinc therapy might have a significant role in reducing the duration of smell and taste recovery, without affecting the total recovery duration from the disease.
Stambouli N et al., 2022 [25]	Zinc	15 mg/day of zinc for 6 weeks	Healthcare workers	A significant decrease in SARS-CoV-2 infection is reported when doxycycline is administered with zinc instead of just the drug.
Rodriguez JAM et al., 2021 [26]	Zinc, Selenium	15 mg/day of Zn and 100 µg/day of Se combined with β-1,3/1,6-glucan for 35 days	General population	Mean levels of CD4+T, CD3+T, and CD8+T cells increase after the second dose of the COVID-19 vaccine due to the intervention. An augmentation in both IgG and IgM is also reported.
Thomas S et al., 2021 [27]	Zinc	50 mg/day of zinc gluconate alone and combined with ascorbic acid for 10 days	COVID-19 ambulatory patients	Treatment with high-dose zinc gluconate alone or combined with vitamin C does not significantly decrease symptoms’ duration compared with standard care.
Abd-Elsalam S et al., 2021 [28]	Zinc	220 mg of Zn sulfate (50 mg of elemental Zn) twice daily	COVID-19 hospitalized patients	Zinc supplementation did not improve the clinical efficacy of hydroxychloroquine.
Tan CW et al., 2020 [47]	Magnesium	150 mg/day of Mg for 14 days maximum, combined with vitamin D and B12	Elderly COVID-19 hospitalized patients	The administration of this oral combination in older COVID-19 patients is associated with a relevant reduction in clinical deterioration requiring oxygen support, ICU support, or both.
Fan L et al., 2021 [48]	Magnesium	Personalized to reach a calcium-to-magnesium intake ratio ≥ 2.6	Population over 65 years old	Twelve-week personalized Mg treatment led to methylation changes in TMPRSS2 gene, confirming another potential role of magnesium intervention in COVID-19 prevention.
Ponikowski P et al., 2021 [51]	Iron	Not specified	Iron-deficient patients stabilized after acute heart failure	Treatment with ferric carboxymaltose decreases the hazard of heart failure hospitalizations, without an apparent effect on cardiovascular death risk
Vinke JSJ et al., 2023 [52]	Iron	1–4 doses of 500 mg of ferric carboxymaltose with six-week intervals	Iron-deficient kidney transplanted patients	Intravenous supplementation efficiently restores iron status, but humoral and cellular immune response against the virus does not improve after three vaccinations.

## Data Availability

Not applicable.
